# Supplementary material for: Does parenting style moderate the relationship between parent-youth sexual risk communication and premarital sexual debut among in-school youth in Eswatini?
Source: PLoS One. 2021 Jan 25;16(1):e0245590. doi: 10.1371/journal.pone.0245590 (PMC7833135; doi:10.1371/journal.pone.0245590)
Supplement: S1 Fig — (DOC) [file pone.0245590.s001.doc]

**Parenting practice**:

Parent-youth sexual risk communication

**Child outcome**:

Premarital sexual debut

**Moderator**:

Parenting style (Authoritativeness)

**S1 Fig.** Conceptual framework. Adapted from Darling and Steinberg’s (1993) contextual model of parenting style.
