# Supplementary material for: Does parenting style moderate the relationship between parent-youth sexual risk communication and premarital sexual debut among in-school youth in Eswatini?
Source: PLoS One. 2021 Jan 25;16(1):e0245590. doi: 10.1371/journal.pone.0245590 (PMC7833135; doi:10.1371/journal.pone.0245590)
Supplement: S2 Fig — (DOC) [file pone.0245590.s002.doc]

F5; n = 116

14 Absent

**487 invited to participate; 479 questionnaires returned; 17 rendered unusable; 462 retained = 95% response rate**

Eswatini: 149 high schools

Manzini region: 56 high schools

Urban = 16 schools

Rural = 40 schools

S1; n = 276

S2; n = 156

5 private + gender-streaming schools

S3; n = 74

F4; n = 137

A B C

A B C

A B C

A B

A A

F4; n = 97

F5; n = 52

F4; n = 29

F5; n = 31

8 Absent

1 refused

7 Absent

**Key**: A,B,C = class streams; F = Form or Grade; n =number of pupils

**S2 Fig. Schematic view of the sampling procedure**
