# Supplementary material for: Does parenting style moderate the relationship between parent-youth sexual risk communication and premarital sexual debut among in-school youth in Eswatini?
Source: PLoS One. 2021 Jan 25;16(1):e0245590. doi: 10.1371/journal.pone.0245590 (PMC7833135; doi:10.1371/journal.pone.0245590)
Supplement: Attachment — Submitted filename: Responses to review comments_17 Sept.docx [file pone.0245590.s007.docx]

PONE-D-19-35873
Does parenting style moderate the relationship between parent-youth sexual risk communication and premarital sexual debut among in-school youth in Eswatini?

Dear Dr Joseph Donlan:

Thank you for your ongoing consideration of our manuscript (PONE-D-19-35873) for publication in the PLOS ONE. We appreciate the time spent by you and the reviewer and believe the revised manuscript is improved. Below, we address the reviewers’ comments.

We look forward to hearing from you regarding our submission. We would be glad to respond to any further questions and comments that you may have.

Sincerely,

Pi-Chen Chang, RN., PhD.

School of Nursing, College of Nursing, Taipei Medical University

250 Wu Hsing Street, Taipei City, Taiwan, 110 R.O.C.

Tel. +886-2-2736-1661

Email: pichen@tmu.edu.tw

| **Comments from the Staff Editor** | |
| --- | --- |
| 1. Please amend your current ethics statement to address the following concerns:  a) Did parents provide their written or verbal informed consent for minors to participate in this study?  b) If consent was verbal, please explain i) why written consent was not obtained, ii) how you documented participant consent, and iii) whether the ethics committees/IRB approved this consent procedure. | Written parental consent was obtained from parents of students younger than 18 years. This has since been stated explicitly on page 14. |
| 2 We note that you have indicated that data from this study are available upon request. PLOS only allows data to be available upon request if there are legal or ethical restrictions on sharing data publicly. For information on unacceptable data access restrictions, please see http://journals.plos.org/plosone/s/data-availability#loc-unacceptable-data-access-restrictions  In your revised cover letter, please address the following prompts:  a) If there are ethical or legal restrictions on sharing a de-identified data set, please explain them in detail (e.g., data contain potentially identifying or sensitive patient information) and who has imposed them (e.g., an ethics committee). Please also provide contact information for a data access committee, ethics committee, or other institutional body to which data requests may be sent. | The authors confirm that, some access restrictions apply to the raw data underlying the findings. Data for the study were collected with the approval of the Eswatini Ministry of Health (MOH)’s National Health Research Review Board (NHRRB). Any researcher who intends to use these data must obtain approval from the NHRRB. To request the data, an individual may either email the lead author (mduyaye@gmail.com) who will facilitate the request with the NHRRB, or contact the NHRRB Secretariat directly at ethicswaziland@gmail.com, or Eswatini Ministry of Health, P.O. Box 5, Mbabane, Eswatini. Otherwise, all pertinent data are within the paper and its Supporting Information files. |
| 3. We note you have included a table to which you do not refer in the text of your manuscript. Please ensure that you refer to Table 2 in your text; if accepted, production will need this reference to link the reader to the Table. | Has since been addressed on page 16. |
| 4. Please upload a copy of Supporting Information S3 and S4 Output files which you refer to in your text on page 25. | Both outputs have since been provided as S3 SPSS Output file and S4 PROCESS macro output file. |
| **Reviewer #1** | |
| 1. I highly commend the authors for conducting a research study that tests the construct of parenting style as a moderator of the relationship between parent-youth sexual risk communication (PYSRC) and premarital sexual debut. Moreover, results from the study further confirm that the construct of parenting style should be considered within cultural context, with attention given to global variability in application or lack thereof. | Thank you. |
| 2. However, based on Baumrind's conceptualization of parenting styles, I would suggest incorporating the other dimensions of authoritarian and permissive (indulgent, neglectful) in investigating the moderating effects of parenting style given the relationship between parenting practices and child development. | The literature review has since been revised to include the four typologies (pages 6 and 7). However, we did not use the four typologies to investigate the moderating effects of parenting style in this study since the instrument (PS II) doesn’t not have scoring instructions for formulating the four parenting style typologies. We also could not locate researchers who had used this scale that way. Instead, we used the methods used by other researchers using this instrument by forming one parenting style (authoritativeness). However, since the PS II has subscales for three parenting style dimensions (i.e. autonomy-granting, responsiveness, and demandingness), we conducted post-hoc analysis for each of these. We will consider the suggestion of four typologies in our future work where we will use tools that can allow us to demarcate the four typologies. |
| 3. Regarding the introduction, more detail could be given on Darling and Steinberg's contextual model of parenting style; however, Baumrind's conceptualizations of parenting style (1971, 1991) should also be noted as part of the theoretical framework. | More details on the contextual model of parenting style has been added as well as Baumrind’s conceptualizations on pages 6. |
| 4. A more thorough review of literature should be presented, highlighting relevant studies that inform the current research and identify significant gaps. | We have since added more relevant literature on pages 7 and 8. |
| 5. Concerning the methodology, the sampling procedures seem to lack academic rigor. The use of random drawing from slips of paper appears to be devoid of sophistication worthy of scientific, empirical investigation. | While the paper method may be less rigorous, it is still considered valid as a sampling strategy among recent literature (Malu, Achinge, Utoo, Kur, & Obekpa, 2020), especially if the sampling frame is small (Gray, Grove, & Sutherland, 2017, page 632), as was the case with the list of schools per strata in our case. |
| 6. Also, more accuracy and detail is needed on the cluster sampling of each grade and the procedure by which individual random sampling is accomplished with each classroom. Two-stage cluster sampling appears to be an appropriate, but accuracy in the application of such a methodological design is lacking. | There was no random sampling at classroom level, instead, all eligible participants were invited to participate. The classroom was the sampling unit in this study. More details on the sampling strategy have since been provided on page 9. |
| 7. Lastly, you should clarify for the readers what is meant by Form 4 and 5. | This has since been clarified on pages 8 and 9. |
| 8. For the discussion, in Lines 270, 277, 318 and 351, the names of authors should be mentioned instead of "studies elsewhere" or "other studies." | This has since been corrected, see pages 17 and 19. |
| 9. Regarding grammatical errors, there are some run-on sentences and lack of appropriately placed semicolons. For example, in Lines 59-63, there are run-on sentences and a need to use semicolons for separation | This has since been corrected, page 4. |
| 10. In Lines 63-66 and Lines 345-347, semicolons are also needed to separate long lists. | This has since been corrected, pages 4, 20 and 21. |
| 11. In Lines 117-118, African-American communities should be placed in parenthesis. | This has since been corrected on page 8. |
| 12. In Line 278, few "had" interactions should be replaced by few "having" interactions. | This has since been corrected on page 17. |
| 13. In Line 337, bias should be used instead of biasness. | This has since been corrected on page 22. |
| **Reviewer #2:** | |
| 1. The authors present the findings as related to “parent-youth sexual risk communication”; however, the sample is comprised of individuals ranging in age up to 24, averaging nearly 19. The sample includes adults and adolescents. Because the measure of sex is whether or not they have ever had sex rather than voluntary debut and age of debut, the question of whether a parent has influence has to be considered. The lower PYSRC scores suggest why authoritativeness has little influence and indicate the need to examine that construct cross-culturally. The lower scores also may be the result of the adult population in which this study was conducted. | This is a fair observation. However, we mentioned in the study that by youth, we mean 15-24 year-olds (page 4), which is the definition of youth used by UN agencies’ e.g. WHO, UNFPA, UNESCO, etc. In Southern Africa, it is not uncommon for these youth to be still in high school, and directly under the care of their parents or guardians. In Eswatini, this is mainly due to some students repeating several grades. For example, in Eswatini, if a pupil fails Grade 12 (the last grade of school), they have to restart at Grade 11 (i.e. they cannot repeat the failed grade (i.e. Grade 12) right away), and same applies for Grade 10, they have to go back to Grade 9, which results in some pupils finishing school later than their counterparts. This was further alluded to in a recent report by the Eswatini Ministry of Education and Training (Government of the Kingdom of Eswatini, 2018).  In addition, in our study, all the participants had never been married and were therefore under their parents’/guardians’ care. In Eswatini, even at university or college, parents/guardians still have an influential role towards their children’s lifestyles or decisions, until they get married or get jobs (financial independence). In fact, the contextual definition of youth in the Eswatini’s strategic and policy documents is “anyone between aged 15-35 years” (Kingdom of Eswatini Ministry of Sports, Culture and Youth Affairs, 2020). That is why is not uncommon to see studies investigating the parental influence even among college students in our region (e.g. Agudile, Okechukwu, Subramanian, Langer, and Geller, 2020). This has also been the case even in the United States (see Hutchinson and Montgomery, 2007 and Bednar and Fisher, 2003).  We should also mention that, actually, during protocol review by the Eswatini IRB, we had initially submitted that we would only include youth aged 15-19 years, however, the IRB members suggested that we open up our age range to 24 years, based on their experience with studies conducted in high schools in the country, especially in rural areas where in-school youth tend to be much older. |
| 2. At the least, the sample should be divided into minors and adults for comparisons before aggregating but a better approach would have been to do that AND use age of debut as the dv. | We have since provided the outputs from the stratified the analysis by age group (minors vs adults) as suggested. S4 PROCESS macro output file.  Regarding the last part of the comment, we appreciate the suggestion, however, that was not part of the objectives of this study. We believe that the suggested analysis if for a different research question altogether, which would require a separate literature review, and updating the methods. That said, we will consider investigating the suggested research question in our future work. Thanks. |
| 3. The findings indicate that authoritativeness did not predict sexual debut. The choice of how to manage parental style was based on Darling’s treatment of her scale; however, you have the ability to determine whether, based on your sample, that was appropriate. Having used Baumrind’s scales in my own work, there is no limitation of the scale in one-parent households and the collinearity problem seems more likely to be an issue with Darling’s scale but that is something the authors can and should test for themselves. | We had actually assessed for the collinearity issue during the previous analysis and we found collinearity only between the demandingness and responsive dimensions (r= .76). However, this was deemed not as an issue in our study since we combined these two dimensions to come up with one parenting style (authoritativeness) in our study. In the revised manuscript, we entered each parenting style dimension in separate models during the post-hoc analysis, of which all dimensions have now been used to test the hypothesis (see S3 Outputs and S4 PROCESS macro output file). |
| 4. The authors also have the ability to conduct post-hoc tests regarding whether the mother or father has more or less influence. | We have since conducted the post-hoc analysis and we found that mothers’ demandingness dimension moderates the association between mother-youth sexual risk communication and premarital sexual debut (see revised discussion section on page 21 and S5 PROCESS macro output file. |

**Cited references**

Agudile, E. P., Okechukwu, C. A., Subramanian, S. V., Langer, A., & Geller, A. C. (2020). Young Adult Sexual Behavior in South Africa: How Important is Parental Social Support? *Afr, J. Reprod Health, 24*(1), 1118-4841. doi:10.29063/ajrh2020/v24i1.5

Bednar, D. E., & Fisher, T. D. (2003). Peer referencing in adolescent decision making as a function of perceived parenting style. Adolescence, 38(152).

Government of the Kingdom of Eswatini. (2018). *National Education and Training Sector Improvement Programme, 2018/19 to 2020/2.* Mbabane, Eswatini. Retrieved https://planipolis.iiep.unesco.org/sites/planipolis/files/ressources/eswatini_netip.pdf

Kingdom of Eswatini Ministry of Sports, Culture and Youth Affairs (2020). *Eswatini National Youth Policy*. Mbabane, Swaziland Retrieved from <https://www.youthpolicy.org/national/Swaziland_2009_National_Youth_Policy.pdf>

Gray, J. R., Grove, S. K., & Sutherland, S. (2017). Burns and Grove's the practice of nursing nesearch: Appraisal, synthesis, and generation of evidence (8th ed.). St. Louis, Missouri: Elsevier, Inc.

Hutchinson, M. K., & Montgomery, A. J. (2007). Parent communication and sexual risk among African Americans. *Western Journal of Nursing Research, 29*(6), 691-707.

Malu, A. O., Achinge, G. I., Utoo, P. M., Kur, J. T., & Obekpa, S. A. (2020). Prevalence of Hepatitis B Surface Antigen and Antibodies to Hepatitis C in the General Population of Benue State, Central Nigeria. *The American Journal of Tropical Medicine and Hygiene, 102*(5), 995-1000. doi:https://doi.org/10.4269/ajtmh.19-0649
