# Supplementary material for: Does parenting style moderate the relationship between parent-youth sexual risk communication and premarital sexual debut among in-school youth in Eswatini?
Source: PLoS One. 2021 Jan 25;16(1):e0245590. doi: 10.1371/journal.pone.0245590 (PMC7833135; doi:10.1371/journal.pone.0245590)
Supplement: Attachment — Submitted filename: Responses to review comments_2 Jan 2021.docx [file pone.0245590.s008.docx]

PONE-D-19-35873
Does parenting style moderate the relationship between parent-youth sexual risk communication and premarital sexual debut among in-school youth in Eswatini?

Dear Dr. Stuart White:

Thank you for your ongoing consideration of our manuscript (PONE-D-19-35873) for publication in PLOS ONE. We appreciate the time spent by you and the reviewers and believe the revised manuscript is improved. Below, we address comments from you and the reviewers.

We would also like to clarify that even though the research did not directly receive any specific grant from funding agencies in the public, commercial, or not-for-profit sectors, the first author (MCS) conducted the study as part of his Master of Science studies which were sponsored by the Taiwanese Government through Taipei Medical University’s International Student Type B Scholarship.

We look forward to hearing from you regarding our submission. We would be glad to respond to any further questions and comments that you may have.

Sincerely,

Pi-Chen Chang, RN, PhD

School of Nursing, College of Nursing, Taipei Medical University

250 Wu Hsing Street, Taipei City, Taiwan 11031, R.O.C.

Tel. +886-2-2736-1661

Email: pichen@tmu.edu.tw

| **Review comments** | **Authors’ responses** |
| --- | --- |
| **Comments from the Academic Editor** | Thank you so much for the positive comments about our manuscript and for the opportunity you have granted us to revise our work.  In retrospect, we acknowledge that the results from the age-stratified analysis were not adequately addressed in the "Results" section in previous drafts. We now present them in the "Results" section on page 16, lines 366~376, and further discuss them on pages 21 and 22, lines 489~501 in the revised (clean) manuscript. Briefly, we are of the view that since none of the age- and gender-stratified analyses showed statistically significant effects, it does not seem that the adults in the sample were suppressing the effect of parenting in the younger sample, which would have been the case had we found significant effects for the younger sample (<18 years of age). The outputs for all the gender- and age-stratified analyses are found in the S3 SPSS Output file and S4 PROCESS macro output file. |
| Before publication, I would ask for two additional revisions, both of which should be minor.  1. Please show that the adults in the sample are not suppressing an effect of parenting in the younger part of the sample. You do reference stratifying the sample by age on page 13- however, the results of this stratification are insufficiently addressed in the results section. Whether you chose to re-run the analyses excluding older subjects, better describe the results of including age as a stratification variable, or address this issue via other means is up to you, as long as the fundamental question is addressed. |  |
| 2. Please address the lack of specific age of sexual debut information as a limitation. It seems highly plausible that sexual behavior in the mid-20's is less related to parenting variables than sexual behaviors in teens. We don't know in the current data, among the older participants, how many participants made their sexual debut in adulthood versus in childhood. I don't feel that this is the fatal flaw that reviewer 2 makes it out to be, but it is worth acknowledging. | We actually have information/a variable on age at sexual debut in the dataset, but we did not initially include it in the current analysis as it was not part of the main objective of the current analysis. However, in the revised manuscript, we now include this variable on page 14, line 339 and in Table 1 on page 15. We also cross-tabulated the current age of the youth against their age at sexual debut to ascertain the proportion of adults who had their sexual debut in adulthood versus in childhood. Briefly, the cross-tabulation revealed that nearly 60% of older youth (18~24 years) had their sexual debut in adulthood. We have now discussed this point on page 21, line 492.  We further performed independent-sample *t*-tests to ascertain if parenting style and parent-youth sexual risk communication significantly differed by current age of the child and their age at sexual debut, and found no significant mean differences. Results of the cross-tabulations and *t*-tests are now mentioned in the "Discussion" section on page 22, lines 503~515 and are presented in the supporting information (see S6 SPSS output file).  Having said that, we should point out the limitation in the measurement of the age-at-sexual-debut variable: participants were asked to choose from predetermined categories of 11 or younger, 12~16, and 17 or older, instead of 18 years or older. Unfortunately, that is how the variable is structured in the US Youth Risk Behavior Survey which is the instrument we adopted for our study, and it was deemed appropriate at the time of data collection since the age of sexual consent in Eswatini was 16 years back then. In summary, as much as it is plausible that sexual behavior in older youth is less likely to be related to parenting variables than in younger youth, that does not seem to be the case in the current sample. One main reason could be that Swati parents do not talk to their children about sexuality issues (as discussed in length in the manuscript), independent of the stage or age of the youth, such that the slope is constant across the adolescent or youth life course. We have presented the above points on page 22. We hope that the information above provides clarity to the issue of parenting and older youth. |
| **Comments from Reviewer #1** | **Authors’ responses** |
| 1. The author(s) addressed all of the comments in the previous round of review. Upon reviewing the manuscript, a second time, I was particularly impressed with the following: (list of comments omitted) | Thank you for the positive feedback regarding our work. |
| **Comments from Reviewer #2:** | **Authors’ responses** |
| 1. The paper provides a very nice discussion of cultural context. This section was particularly well done. | Thank you for the positive feedback. |
| 2. In their response to reviewers, they say they “addressed the reviewers’ comments one by one.” A more common and helpful mode of response to reviewers is to actually list the comments and show how you addressed them. | We actually did that in our first revision. We listed each comment from both reviewers in a table with two columns: one with the comments and the other stating how each comment was addressed. |
| 3. Age of debut was not discussed. I'd suggest you offer that as a question for future research. The decision, then seems to boil down to whether a paper of non-significant findings makes a contribution. In my opinion, given the fundamental question of adults and parental influence, I do not see enough value, I'm sorry to say. Had the dv been age of debut, perhaps, which is a shame because the paper is very well written. | We actually have information/a variable on age at sexual debut in the dataset, but we did not initially include it in the current analysis as it was not part of the main objective of the current analysis. However, in the revised manuscript, we now include this variable on page 14, line 339 and in Table 1 on page 15. We also cross-tabulated the current age of the youth against their age at sexual debut to ascertain the proportion of adults who had their sexual debut in adulthood versus in childhood. Briefly, the cross-tabulation revealed that nearly 60% of older youth (18~24 years) had their sexual debut in adulthood. We have now discussed this point on page 21, line 492.  We further performed independent-sample *t*-tests to ascertain if parenting style and parent-youth sexual risk communication significantly differed by current age of the child and their age at sexual debut, and found no significant mean differences. Results of the cross-tabulations and *t*-tests are now mentioned in the "Discussion" section on page 22, lines 503~515 and are presented in the supporting information (see S6 SPSS output file).  Having said that, we should point out the limitation in the measurement of the age-at-sexual-debut variable: participants were asked to choose from predetermined categories of 11 or younger, 12~16, and 17 or older, instead of 18 years or older. Unfortunately, that is how the variable is structured in the US Youth Risk Behavior Survey which is the instrument we adopted for our study, and it was deemed appropriate at the time of data collection since the age of sexual consent in Eswatini was 16 years back then. In summary, as much as it is plausible that sexual behavior in older youth is less likely to be related to parenting variables than in younger youth, that does not seem to be the case in the current sample. One main reason could be that Swati parents do not talk to their children about sexuality issues (as discussed in length in the manuscript), independent of the stage or age of the youth, such that the slope is constant across the adolescent or youth life course. We have presented the above points on page 22. |
